# Identification of *Coxiella burnetii* in Raw Milk of Livestock Animal in Iran

**DOI:** 10.1155/2021/6632036

**Published:** 2021-01-18

**Authors:** Ashraf Mohabati Mobarez, Ehsan Mostafavi, Mohammad Khalili, Saber Esmaeili

**Affiliations:** ^1^Department of Bacteriology, Faculty of Medical Sciences, Tarbiat Modares University, Tehran, Iran; ^2^Department of Epidemiology and Biostatics, Pasteur Institute of Iran, Tehran, Iran; ^3^National Reference Laboratory of Plague, Tularemia and Q Fever, Research Centre for Emerging and Reemerging Infectious Diseases, Pasteur Institute of Iran, Akanlu, Kabudar-Ahang, Hamadan, Iran; ^4^Department of Pathobiology, Faculty of Veterinary Medicine, Shahid Bahonar University of Kerman, Kerman, Iran

## Abstract

*Coxiella burnetii* is the causative agent of Q fever in humans and animals. This study aimed to determine the frequency of *C. burnetii* in milk samples of dairy animals (goats, sheep, and cattle) in some selected regions in Iran, where there is no information about prevalence of *C. burnetii*. In this study, 162 individual milk samples were collected from 43 farms in three provinces (Tehran, Hamadan, and Mazandaran). Real-time PCR was used for the detection of IS1111a element of *C. burnetii*. In total, 23 of 162 samples (14.2%, 95% conﬁdence interval (CI): 9.65–20.2%) were positive for *C. burnetii* by real-time PCR. *C. burnetii* was detected in 10.17% (95% CI: 4.74–20.46) of goat milk samples. In sheep milk samples, 18.6% (95% CI: 9.74–32.62) were positive, and *C. burnetii* was detected in 15% (95% CI: 8.1–26.11) of cattle milk samples. Molecular evidence of the presence of *C. burnetii* was seen in milk samples of dairy animals in all the studied regions. These findings demonstrated that *C. burnetii* infection, especially in raw milk samples, deserves more attention from the health care system and veterinary organization in Iran.

## 1. Introduction

*Coxiella burnetii* is a fastidious and obligate intracellular bacterium which is the causative agent of Q fever in humans and animals. *C. burnetii* is a very infective organism and highly resistant to harsh environmental conditions such as physical and chemical stresses [1].

Q fever is a worldwide distributed zoonosis in which clinical signs vary from flu-like symptoms for acute Q fever (self-limited disease) to serious endocarditis for chronic Q fever in humans [2]. Inhalation of *C. burnetii*-infected aerosols is the most frequent route of transmission to humans [1]. Farm animals including cattle, sheep, and goats are the main reservoirs of *C. burnetii* [2]. Coxiellosis is often asymptomatic in livestock, but clinical manifestations such as abortion, stillbirth, infertility, mastitis, and endometritis have been reported [3, 4]. Infected farm animals shed high numbers of *C. burnetii* in birth products, milk, faeces, vaginal mucus, and urine [1]. Shedding of *C. burnetii* can persist for several months up to 1-2 year in vaginal mucus, faeces, and milk, after initial infection [5].

Given the high prevalence of this bacterium in farm animals, it is necessary to evaluate the presence of *C. burnetii* in food of animal origin. Milk is an important source of *C. burnetii* among the foods of animal origin [6]. Infected livestock shed *C. burnetii* into milk for variable periods. Their milk can become contaminated with this bacterium through faecal maters, vaginal mucus, or urine [7]. Ingestion of contaminated milk or dairy products can thus be a source of infection in humans. The significant prevalence of this bacterium in milk has led to increased concern about the role of dairy products as a possible infection source of Q fever disease in humans [6].

Based on current data, Iran is considered endemic for Q fever, and this disease has significant seroprevalence among animal and human populations [8]. However, human clinical cases of Q fever are rarely diagnosed and reported by the Iranian health care system. Based on a systematic review and meta-analysis study in Iran, pooled estimated prevalence of *C. burnetii* in milk was 15.1%, 7.8%, and 3.8% in the milk of cow, goat, and sheep, respectively [7]. Despite several studies about the prevalence of *C. burnetii* in milk samples in Iran, there is still no information about infection spreading in most parts of the country. The present research aimed to detect *C. burnetii* presence in milk samples in less-investigated regions of Iran including Tehran, Hamadan, and Mazandaran provinces.

## 2. Materials and Methods

### 2.1. Sampling

**Figure 1:**
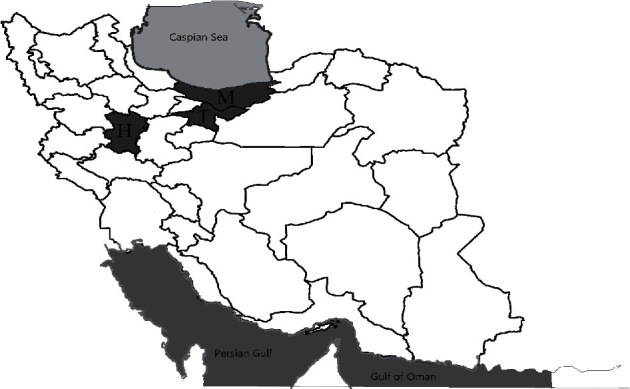
Sampling area in three provinces of Iran: Tehran (T), Mazandaran (M), and Hamadan (H).

Sampling was carried out from June to August 2017 in Tehran, Mazandaran, and Hamadan provinces (Figure 1). Sampling was performed from dairy animal farms raising goats, sheep, and cattle. From each individual dairy animal, 50 mL raw milk was collected in a sterile tube and according to protocols approved by Ethics Committee of Biomedical Research of Tarbiat Modarres University (ethic code: IR.TMU.REC.1395.510).

### 2.2. Milk Processing and DNA Extraction

All milk samples were processed in the laboratory to remove of cream and precipitation cells (included bacteria) based on previously published protocols [9]. The final precipitate was dissolved in 1 mL phosphate‐buffered saline (PBS) solution and preserved at −20°C until DNA extraction. An aliquot of the sediment solution (200 *μ*L) was used for DNA extraction. Roche High Pure PCR Template Preparation Kit (Roche, Germany) was used for genomic DNA isolation. All extraction steps were done according to the manufacturer's instruction.

### 2.3. Molecular Identification of *C. burnetii*

Real-time PCR (TaqMan Real-time PCR) was performed using primers (forward; AAAACGGATAAAAAGAGTCTGTGGTT, reverse; CCACACAAGCGCGATTCAT) and probe (6-FAM-AAAGCACTCATTGAGCGCCGCG-TAMRA) targeting IS1111a element of *C. burnetii* [10]. Each real-time PCR reaction (final volume 20 *μ*L) included 900 nM of each forward and reverse primers, 200 nM probe, 10 *μ*L of 2x RealQ Plus Master Mix for Probe (Ampliqon, Denmark), and 4 *μ*L of extracted DNA. The real-time PCR program was 95°C for 10 minutes, followed by 94°C for 15 seconds and 60°C for 60 seconds (45 cycles) in a Corbett 6000 Rotor-Gene system (Corbett, Victoria, Australia). DNA of Nine Mile strain (Nine Mile Phase I/RSA 493) was used as a positive control, and double distilled water was used as a negative control. Results were analyzed using Rotor-Gene® *Q* 2.3.5 software (QIAGEN). Samples were considered positive in real-time PCR analysis, if their cycle threshold (Ct) values were 38 or lower.

### 2.4. Data Analysis

Data analysis was performed using SPSS software (version 24, SPSS Inc., Chicago, IL). *P* values lower than 0.05 were considered significant.

## 3. Results

In total, 162 milk samples were collected from 43 farms in three provinces (Tehran, Hamadan, and Mazandaran). Fifty-nine samples (from 14 dairy goats' herds) were collected from Tehran and Mazandaran provinces (Table 1). In addition, 43 sheep milk samples and 60 cattle milk samples were collected from thirteen and sixteen dairy herds, respectively (Tables 2 and 3).

In total, 23 of 162 samples (14.2%, 95% confidence interval (CI): 9.65–20.2%) were positive for *C. burnetii* by real-time PCR. *C. burnetii* was detected in 10.17% (95% CI: 4.74–20.46) of goat milk samples. The prevalence of *C. burnetii* in Mazandaran and Tehran provinces was 11.11% and 10%, respectively (Table 1).

*C. burnetii* was detected in 18.6% (95% CI: 9.74–32.62) of sheep milk samples. The lowest and highest prevalence of *C. burnetii* were in Hamadan (10.53%) and Tehran (33.33%) provinces, respectively (Table 2).

Finally, *C. burnetii* was detected in 15% (95% CI: 8.1–26.11) of cattle milk samples. The highest rates of *C. burnetii* were seen in Tehran (23.08%) and Hamadan (15%) provinces, respectively. No positive sample was found in cattle milk samples of Mazandaran province (Table 3).

## 4. Discussion

This study was conducted in less-investigated regions of Iran with no previous data on *C. burnetii* infection in animals and raw milk. Based on our results, molecular evidence of *C. burnetii* was detected in milk samples of dairy animals in all studied regions. Various studies in Iran show that Q fever is a challenge in Iran. In our study, 14.2% of raw milk samples were positive for *C. burnetii* by real-time PCR. These findings demonstrated that *C. burnetii* infection, especially in raw milk samples, should be a detection target for veterinary organization and the health care system in Iran.

Based on the results of this study, *C. burnetii* was detected in 10.17% of goat milk samples by real-Time PCR. The prevalence of *C. burnetii* in Mazandaran and Tehran provinces was 11.11% and 10%, respectively. Different prevalence rates of *C. burnetii* in goat milk were reported from Iran: 16.6% in West-Azerbaijan province (northwest of Iran) [11], 16.1% in Kerman province (southeast of Iran) [12], 1.8% in Chaharmahal-va-Bakhtiari province (southwestern of Iran) [13], and 4.5% in Isfahan province (central of Iran) [14]. Also, a high prevalence (35.7%) of *C. burnetii* was reported in milk samples from goats with abortion history in Qom province (central Iran) [9]. In other countries, presence of *C. burnetii* in goat milk was reported, namely, 6.3–12.1% in Belgium [15], 14.3% in USA [16], and 17.2% in Lebanon [17]. Goat's milk is usually consumed in nonpasteurized form in some countries. Also, no temperature and pasteurization process are used to produce dairy products from goat milk. Therefore, serious attention must be paid to the presence of *C. burnetii* in goat's milk.

In the current study, 18.6% of sheep milk samples were positive for the causative agent of Q fever. The prevalence of this bacterium in sheep milk was also 33.3%, 20%, and 10.5% in Tehran, Mazandaran, and Hamadan provinces, respectively. In other studies, the prevalence of *C. burnetii* in sheep's milk was reported to be 7.6% in West-Azerbaijan province [11], 5.7% in Isfahan province [14], 20.8% in Lorestan province (western of Iran) [18], 3.3% in Zanjan province (northwest of Iran) [19], and 35.7% in Qom province [9]. The prevalence in other countries was 10% in Lebanon [17], 4% in Hungary [20], 6.5% in Turkey, [21] and 22% in Spain [22]. According to the findings of this study and other studies in Iran and other countries, it seems that *C. burnetii* is very common in sheep's milk. Like goat milk, there is a strong interest to consume raw sheep's milk and its products in Iran, especially in rural and nomadic population that consume these unpasteurized. Therefore, it is very important to pay attention to the milk-borne pathogens in such communities, and the veterinary organization must prioritize control and prevention strategies in livestock. The health care system should also provide training for at-risk people.

Fortunately, cow's milk is mostly consumed in pasteurized form in Iran, and there is less interest in consuming cow raw milk and its products in unpasteurized form. However, in some rural and underdeveloped areas, there is still a tendency to consume unpasteurized raw milk and its products. *C. burnetii* was detected 15% in cattle milk samples in this study. The prevalence of *C. burnetii* was 23.1% in Tehran, 15% in Hamadan, and 0% in Mazandaran provinces. Based on recent studies, the prevalence of *C. burnetii* was reported to be 8.6% in Isfahan province [23], 33.3% in Qom province [9], 8.3% in Zanjan province [19], and 11% in Fars province (south of Iran) [24]. Different prevalence rates of *C. burnetii* were reported among cattle milk from other countries: 8.7% in Hungary [20], 15.1% in Lebanon [17], 18.8% in the Netherlands [25], and 27% in Italy [26]. Therefore, shedding in milk by bovines is the most important route of spreading this bacterium in the environment in all investigated countries.

One of the limitations of this study was the small number of samples in some areas, which made it difficult to judge the prevalence comparison. Therefore, it is suggested that a large number of samples be collected from the study areas in future studies.

Based on our results, molecular evidence of *C. burnetii* was detected in milk samples of dairy animals in all studied regions. These findings demonstrated that *C. burnetii* infection, especially in raw milk samples, could pose a serious risk of Q fever to farmers and consumers in Iran.

## Figures and Tables

**Table 1:** Presence of *C. burnetii* in caprine milk samples in study regions by real-Time PCR in 2017-2018.

	Number of sampled herds	Number of samples	Number of positive samples	Rate of positive samples (95% CI)
Tehran	11	50	5	10 (4.35–21.36)
Mazandaran	3	9	1	11.11 (1.99–43.5)
Total	14	59	6	10.17 (4.74–20.46)

**Table 2:** Detection of *C. burnetii* in sheep milk samples by Real-Time PCR in 2017-2018.

	Number of sampled herds	Number of samples	Number of positive samples	Rate of positive samples (95% CI)
Tehran	3	9	3	33.33 (12.06–64.58)
Mazandaran	5	15	3	20 (7.05–45.19)
Hamadan	5	19	2	10.53 (2.94–31.39)
Total	13	43	8	18.6 (9.74–32.62)

**Table 3:** Presence of *C. burnetii* in cattle milk samples in some Iranian provinces in 2017-2018.

	Number of sampled herds	Number of samples	Number of positive samples	Rate of positive samples (95% CI)
Tehran	2	13	3	23.08 (8.18–50.26)
Mazandaran	2	7	0	0 (0–35.43)
Hamadan	12	40	6	15 (7.06–29.07)
Total	16	60	9	15 (8.1–26.11)

## Data Availability

The data that support the findings of this study are available from the corresponding author upon reasonable request.
